# Psychological Distress of International Students during the COVID-19 Pandemic in China: Multidimensional Effects of External Environment, Individuals’ Behavior, and Their Values

**DOI:** 10.3390/ijerph18189758

**Published:** 2021-09-16

**Authors:** Tao Xu

**Affiliations:** College of Law and Political Science, Zhejiang Normal University, Jinhua 321004, China; xutao@zjnu.edu.cn

**Keywords:** formal social support, collectivist values, individual values, COVID-19, psychological distress

## Abstract

The COVID-19 epidemic has had a significant impact on society. In particular, it has had a strong impact on college students, including international students. Through an online questionnaire survey, it is found that the psychological distress experienced by international students is the result of a combination of the external environment (including the lockdown measures, social distancing, and social support) and internal factors such as values and behavior. The analysis shows that the new teaching mode and the corresponding changes in learning behavior are significantly associated with the psychological distress brought about by the COVID-19 epidemic. In addition, the influence of international students’ values also plays a significant role in their psychological distress. Collective values are conducive to the alleviation of psychological distress, while individual values have the opposite effect. At the same time, the study also reveals that if there is sufficient social support, isolation (due to lockdown or social distancing early or later on) is not necessarily directly related to psychological distress. However, only formal social support can effectively alleviate psychological distress, while informal social support does not play a similar role. These conclusions have certain policy significance for the prevention of and response to epidemics in other countries.

## 1. Introduction

The outbreak of COVID-19 in Wuhan has spread rapidly worldwide. Almost 215 million people have contracted COVID-19 according to World Meters’ real-time data (https://www.worldofmeters.com, accessed on 4 September 2021), which has led to serious economic and social consequences worldwide [1,2,3,4,5,6,7,8,9]. Simultaneously, the COVID-19 pandemic not only affected physical health, but also mental health and well-being [10]. Nearly one in three to six people appear adversely affected by depression, anxiety, insomnia, or suicidal ideas [10,11,12].

In response to the COVID-19 epidemic, various countries have adopted similar strategies such as lockdown or quarantine, etc. The pandemic and the related containment measures including quarantine, social distancing, and self-isolation can have a detrimental impact on mental health [12]. Regarding the impact of lockdown on the general population, previous studies have shown that among the unaffected population in China, the proportions of anxiety and distress are 8.3% and 14.6%, respectively [13], and the proportions of moderate to severe stress, anxiety, and distress are 6.5–8.1%, 28.8%, and 16.5% [14]. This did not slow down significantly over time [15]. Additionally, the mental health of college students was significantly affected during public health emergencies, and they required attention, help, and support from society, their families, and their colleges [16]. Another study in Italy showed that with a long time being in a lockdown situation, there is a very significant increase in the level of depressive, anxiety, and stress symptoms [17]. To some extent, both lockdown and quarantine can be associated with isolation. Some studies have examined the psychological distress resulting from the pandemic [10,18,19]. From them, we will find that almost all lockdown, quarantine, or social distancing measures that cause psychological distress have the same characteristics—that is, individuals’ social support becomes reduced or even lost completely. A large amount of previous literature has revealed that social support networks can significantly reduce people’s psychological distress [20,21,22,23]. Long-term lockdown is likely to change people’s social interactions, thereby reducing mutual emotional and material support.

For students, approximately one-quarter of college students experienced anxiety during the COVID-19 epidemic. Students’ economic stressors, effects on daily life, and academic delays were positively associated with the level of anxiety symptoms [16]. In China, during the pandemic, all primary and middle schools were closed, and universities either postponed their classes or used online learning methods. These measures had a specific impact on education [6]. Some studies have revealed the negative relationship between internet use and psychological distress [24,25]. Increased participation in inappropriate internet-related activities among students was associated with greater psychological distress. The logic behind such research is that a preoccupation with online activities could prevent an individual from engaging in normal socializing behaviors and cause psychological distress [26].

Generally, there seems to be no connection between people’s values and their psychological distress. However, studies show that values are closely associated with psychological distress. For instance, some studies revealed that value-based actions are often associated with improved mental health [27]. Having values that prioritize enduring active challenges, cherishing family and friends, and a commitment to values in adolescence may reduce psychological distress in adults [28].

Existing studies have explored the negative consequences of the COVID-19 epidemic for ordinary residents’ mental health [14,16,19,29,30,31,32,33,34], but little attention has been paid to the impact of COVID-19 on international students in terms of psychological distress and associated factors. International students are one of the most affected groups. On the one hand, they are far away from their home country and relatives, with reduced social support. On the other hand, they are easily ignored in the face of sudden epidemics, especially when the destination country is also in crisis.

In addition, previous studies have shown that age, gender, income, and social status are all closely related to psychological distress among individuals [35,36,37]. Other studies have revealed how stimulus factors and sudden changes in the external environment, such as the pandemics resulting from SARS (Severe Acute Respiratory Syndromes), MERS (Middle East Respiratory Syndrome Coronavirus), and COVID-19, etc., lead to psychological distress [13,29,32,35,38,39]. This implies that psychological distress is the confounding result of multiple factors. Many psychological analyses have examined psychological problems such as panic, fear, distress, and PTSD (Post-Traumatic Stress Disorder) that emerge during or after emergencies, incidents, disasters, and epidemics, etc. [40,41,42,43,44]. However, there are comparatively fewer non-psychological and social-related analyses. Multidimensional analysis posits that any person exists within a multidimensional system involving biology, psychology, and society, which interact. According to this, a person’s psychological state is related to the environment, society, and behavior as well [45]. Therefore, the psychological distress experienced during the epidemic period is expected to be the result of combined effects of the external environment, people’s behavior, and their values.

**Figure 1:**
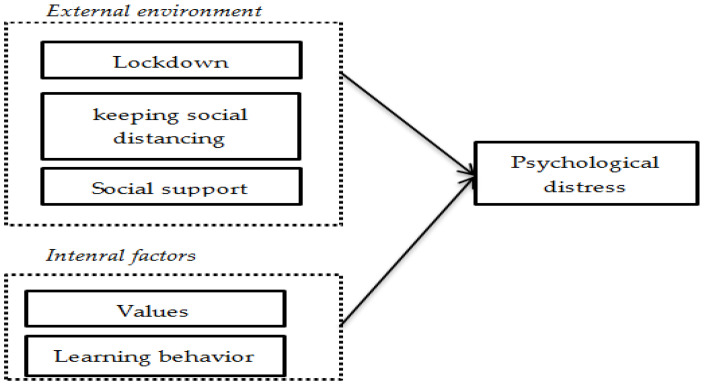
Analytical framework of international students’ psychological distress.

This article will attempt to overcome the shortcomings of the single psychological perspective that emerged in the past, and then analyze the social and cultural factors associated with psychological distress by a new analytical framework (Figure 1). In this framework, the psychological distress of international students is the result of a combination of the external environment (including lockdown, social distancing, and social support) and internal factors such as values and behavior. This will contribute to a more comprehensive understanding of the causes of psychological distress from multiple perspectives.

According to this analytical framework, we have the following inferences:

Firstly, lockdown or social distancing does not necessarily reduce people’s social support, because people—especially international college students, who are proficient in using the internet—can still maintain close relationships through online communication, which can provide better social support. Moreover, during the COVID-19 pandemic, governments and organizations at all levels have taken unified actions, including closing communities, persuading ordinary people to maintain social distancing, ensuring the adequate distribution of supplies, and providing daily services for quarantined individuals, such as meals, temperature testing, drug distribution, and other services. During this period, although the informal social support received by individuals has been affected to a certain extent, the official social support received from the government and organizations has been increased. Therefore, we propose:

**Hypothesis** **1.** *As long as people have sufficient material and emotional support, quarantine or social distancing is unlikely to cause them severe psychological distress.*

Secondly, collectivist values and individualistic values play different roles in the psychological distress of international students. On the one hand, in order to implement measures in a short time, the government requires the understanding and support of its citizens. These urgent measures are aimed at protecting the interests of the group and may impinge on the interests and freedom of the individual. If people uphold collectivist values, they can fully understand and support them. On the contrary, if they uphold their individualist values, they may doubt or even resist, delaying the process of taking emergency action, thus losing opportunities and leading to negative effects on a larger scale. On the other hand, people with liberal values believe that the actions of the collective or the organization will inevitably impinge on their individual interests, and they do not believe in the collective and the organization. Then, we propose:

**Hypothesis** **2.** *Collectivist values are conducive to alleviating psychological distress, while individualistic values are just the opposite.*

Thirdly, the new teaching model places economic pressure on international students. Online teaching requires students to purchase computers, tablets, or smartphones and install teaching software, requiring a faster network speed. However, many international students are often poor. They mainly rely on scholarships provided by the Chinese government to live and study in China. The COVID-19 epidemic interrupted normal life and study. In order to continue their studies, students had to purchase equipment and broadband with a faster transmission speed, which may have been expensive.

Even if they can afford the cost of learning equipment and internet service, the need to learn how to use the new teaching software may still cause considerable pressure and distress among students. The software used by Chinese universities is almost entirely developed by Chinese companies. They need to spend a great deal of extra time learning how to use the software, increasing the difficulty of their already challenging courses. Moreover, compared with the traditional classroom teaching and learning model, the new online teaching and learning model involves less interaction or lower communication efficiency between teachers and students, which reduces students’ learning efficiency and hinders international students’ acquisition of knowledge to a certain extent. We propose:

**Hypothesis** **3.** *There is a significant positive correlation between the economic pressure of online learning* *, adaptation to online classes, and students’ distress.*

The current study explored (1) the type of social support required to prevent the psychological distress arising due to the lockdown and social distancing measures; (2) the relationship between values and psychological distress among international students; and (3) the relationship between learning behavior and psychological distress among international students.

## 2. Method

### 2.1. Data Collection and Quality Control

The survey was conducted at a provincial university “Z” in Eastern China’s Zhejiang Province. This is a comprehensive university with nearly 26,000 full-time students, including a particularly large number of international students, who come from more than 60 countries on five continents. Due to the extensiveness and heterogeneity of the student population, it is considered appropriate to conduct surveys of international students. In order to study the psychological factors affecting international students during the COVID-19 epidemic, we conducted a questionnaire survey from 15 to 31 August 2020. In this survey, we asked the international students to answer a set of questions about their psychological symptoms and behaviors during and after good control of the COVID-19 epidemic in China. This survey was approved by the university research department office and supported by the international students’ office after ethical consideration (Research ethics committee investigated the ethical issues that might be involved in the research, such as whether the respondent had the right to voluntarily answer or refuse to answer, whether the questionnaire involved the respondent’s personal privacy, what information would be collected and how they would be protected, whether the respondents would have an informed consent letter before filling in the questionnaire, whether the informed consent letter contained information such as the purpose of the investigation and measures to protect their information). Since many international students had graduated and new students had not been registered before the beginning of the survey, there were only approximately 1200 registered international students in the 18 teaching schools of the university at the time of the survey. Therefore, we recruited volunteers among these students only. Specifically, in this survey, the relevant staff from the international office and international student counselors posted the questionnaire to the working WeChat group of international students. Questionnaires in 3 languages were provided, namely Chinese, Russian, and English. The international students volunteered to fill in the questionnaire; before they started, a consent letter was provided to inform them of the purpose of the survey and that all information provided would be strictly protected according to the law. Finally, 351 questionnaires were obtained. To ensure the quality of the questionnaire survey, we set three quality control questions in the questionnaire (for example, which of the following pictures is the Great Wall? 3 plus how much is equal to 6?) (Since the international students had received more than 1 year’s training on Chinese language and culture on average, nearly all of them knew about the Great Wall, Therefore, there is no need to worry about someone being ruled out because they don’t know the Great Wall). Only when all three questions were correctly answered could they be regarded as qualified and effective questionnaires. After checking, only 289 questionnaires were considered to be valid and finally used as the data for analysis.

### 2.2. Participants

Originally, 351 participants were included; after strict screening according to the set selection standard, the results of 62 participants were eliminated because of the lack of complete and right answers to the three quality control questions. The present study finally included 289 participants (61.25% male, mean age = 24.38 years, SD = 4.41). They came from more than 60 countries around the world.

### 2.3. Measures

#### 2.3.1. Dependent Variable

In the questionnaire, a set of subjective evaluation variables that scale psychological distress [46,47] were included, such as feeling so sad that nothing could cheer you up, feeling nervous, feeling so restless that you could not sit still, feeling hopeless, feeling that everything was an effort, feeling worthless, etc. (The Kessler distress scales are extensively used around the world, including in China. Previous studies have confirmed their validity and reliability (Benjamin et al. 2010; Furukawa et al. 2003; Nian 2018; Zhang et al. 2010; Xu et al. 2013)). The alternative answers ranged from very inconsistent to very consistent by 5 intervals, which were coded as 1 to 5. We then tested the correlations among the indicators in this emotional variable group and found that the indicators were closely related. The reliability test indicated good reliability (Cronbach’s alpha = 0.9089), and the validity was also confirmed to be good in extensive previous research [48,49,50,51,52]. We summed the 6 items and took the sum score as the dependent variable, psychological distress.

#### 2.3.2. Independent Variable

##### Explanatory Variables

*Lockdown.* We included a question asking whether the community or school was closed, and it was treated as a binary variable in the analysis: yes is coded as 1; no is coded as 0.

*Time to start social distancing.* Respondents were asked in the questionnaire in which month they started to adhere to social distancing guidelines. According to the respondents’ answers, the author sorted the answers and then coded them as numbers 1–8, where the smaller the number, the earlier the respondent started social distancing, and the larger the number, the later they begun social distancing. It was coded as an ordinal variable.

*Social support.* A group of questions in the questionnaire inquired about the social support received by the respondents during the epidemic period. They specifically asked about the kind of support that was provided by their family members, relatives, or classmates in the same country, international students or friends from other countries, Chinese friends or classmates, schools, and the teachers at their university. The alternative answers were divided into providing materials, information, or substantial support for learning; emotional support such as care and comfort; and support both on a material and emotional level. We coded the answers as follows: providing no support as 0, providing either material or emotional support as 1, and providing two kinds of social support as 2. This was treated as an ordinal variable in the analysis. From the perspective of categories, social support is usually divided into informal social support and formal social support. The former usually comes from relatives, friends, or classmates, while the latter usually comes from formal social organizations or institutions [23]. In this sense, among foreign students, support from schools or teachers can be regarded as formal social support, and other types can be regarded as informal social support.

*Values.* Values can be defined as broad preferences concerning appropriate courses of action or outcomes. As such, values reflect a person’s sense of right and wrong or what “ought” to be [53]. There were 2 questions in the questionnaire focusing on the respondents’ views on personal and collective interests. The specific questions were “how do you agree with the view that personal interests should take precedence over collective interests” and “Individuals should be more important than the team”. The alternative answers ranged from strongly disagree to strongly agree, with 4 categories. We coded them 1 to 4, and then summed the two as the score of the values. These two expressions carry an intrinsic tension between individualism and collectivism. The more a respondent agreed with them, the stronger their individualistic values, and the less they agreed with them, the stronger their collective values.

*Learning.* The learning situation included three aspects. The first was to ask whether participating in online learning increases the financial burden of the family. The alternative answers were, respectively, “a decrease, no increase, and an increase”. We assigned the responses a value of 1 to 3. The second was to ask about the fluency of the online learning network: the alternative answers were very good, relatively good, fair, relatively poor, and very poor, and the answers were assigned values from 1 to 5, respectively. The third asked whether they had adapted to the online teaching methods, and the possible answers were very adapted, relatively adapted, general, not very adapted, and not adapted at all, which were assigned values of 1 to 5, respectively.

##### Control Variables

*Length of stay in China.* This question inquired about the length of the respondent’s stay in China and they provided the answer in terms of the number of months that they had stayed in China, which was treated as a continuous variable.

*Sex* is a dichotomous variable; we coded male as 0 and female as 1. *Age* is a continuous variable, computed from the birth-year variable, specifically calculated by the survey year minus the birth year. It was re-coded into a categorical variable during analysis, divided into four groups: below 22 years old, 23–30 years old, 31–36 years old, 37 years old and above.

### 2.4. Data Analysis Strategy

This study used the linear regression equation model [54,55] and the OLS (ordinary least squares) method [56] to analyze public psychological distress. The specific expression is as follows:

Y = β_0_ + β_1_X_1_ + β_2_X_2_ + …… + β_k_X_n_ + ε

Y is the dependent variable of psychological distress, X is the various independent variables that may affect psychological distress, and β is the relative influence coefficient.

In order to test the robustness of the model results, we reconstructed the dependent variable again using exploratory factor analysis and then rebuilt all the models. By comparison between the models’ results, we tested their robustness.

## 3. Results

### 3.1. Descriptive Statistics

From Table 1, we can see that there were 177 males in the sample, representing approximately 61.25%, and 112 females, representing approximately 38.75%. In terms of age distribution, 36.33% were under 22 years old, 55.36% were 23 to 30 years old, 7.27% were 31 to 36 years old, and only 1.04% were over 37 years old. In total, 94.81% of people reported experiencing a lockdown. At the same time, 7.61% of the people reported that the running of online classes was very smooth, 26.3% of people reported that it was smooth, 28.72% reported that it was general, 16.61% reported that it was occasionally stuck, and 5.19% reported that it was always stuck. Meanwhile, for the new teaching model of online courses, only 11.76% of people said that they were fully adapted, 24.57% reported that they were more adapted, 30.45% reported that they were generally adapted, 15.92% reported that they were not quite adapted, and 1.73% reported they were not adapted. Regarding the question of whether the new teaching model had placed economic pressure on their families, only 2.46% of people said that it had not, while 40.57% of people said that it had placed some pressure on their families. Lastly, students had spent an average of nearly 27 months in China.

To estimate the international students’ psychological distress during the COVID-19 epidemic, we summed the six items used as indicators of psychological distress and found that the mean sum value is approximately 13.97 (Table 1), less than half of the total score, which means that although international students experienced a certain degree of psychological distress, it was not severe (According to the psychological distress scales, a higher the sum score indicates serious psychological distress, and a lower sum score indicates less psychological distress. The mean sum score was less than half the sum score, which clearly shows that the students experienced some distress but it was not serious).

Furthermore, in Table 1, due to the Chi 2 test results, we can see that sex, age group, and lockdown were not closely related to psychological distress (*p* > 0.05). Meanwhile, values, network smoothness, adaptation to the new learning model, and economic pressure were closely related to psychological distress (*p* < 0.05).

Simultaneously, in order to explore the possible relationships between the scale variables and psychological distress, such as support from their own and other countries, support from Chinese classmates and friends, and support from the university and faculties, we performed correlation analysis. The results show that the time spent in China, the time at which social distancing started, support from their own country, support from other countries, and support from Chinese classmates and friends were not correlated with international students’ psychological distress, while support from the university and faculties, low network smoothness, adaptation to online classes, economic pressure, and values were very significantly correlated with psychological distress among the students (Table 2).

### 3.2. Regression Results

In order to distinguish the influences of multiple factors such as individual learning behaviors, values, and external environment on the psychological distress experienced by international students, we constructed four models. Model 1 examined the influence of the external environment (including lockdown and social distancing measures) on psychological distress, and Model 2 examined the influence of social support behind the environment on psychological distress. Meanwhile, Model 3 tested the influence of values, and Model 4 tested the influence of individual learning behaviors on the psychological distress experienced by international students.

In Table 3, the results of Model 1 show that community lockdown did not necessarily lead to psychological distress. The closure of the community had no significant impact on international students’ psychological distress. The spread of COVID-19 may have been hindered to some extent by maintaining social distancing, but beginning social distancing earlier or later had no significant effect on mental status, especially on psychological distress.

To explore the social support received by the students, we constructed Model 2. The results of the model show that, on the whole, social support could effectively reduce the distress experienced by international students, but not all social support had such a positive effect—only specific kinds of social support could produce a positive effect. Specifically, the social support network of the student’s home country, including the social support of their family members, relatives, and friends in the same country, could not effectively alleviate students’ distress. In addition, substantive help such as material, information, learning, as well as emotional support, such as care and sympathy, could not effectively alleviate their distress caused by COVID-19. The material support and emotional support of international students or friends from other countries also could not effectively alleviate the participants’ distress; only the material and emotional support provided by the school and teachers at their university or college could effectively alleviate the distress caused by the epidemic (β = −1.841; *p* < 0.05).

The material or emotional support provided by the former two belong to the informal level, while the latter belongs to the formal level. Therefore, we can see that the positive role of the formal level of social support may have been greater than that of the informal level during the course of the COVID-19 epidemic. This shows that Hypothesis 1 is confirmed.

The results of Model 3 show that values significantly affected international students’ distress. Specifically, people who upheld individualistic values were more likely to be distressed (β = 1.076; *p* < 0.001), while, simultaneously, it is implied that people who uphold collectivist values are less likely to be distressed. This means that Hypothesis 2 is confirmed.

The results of Model 4 show that there was a significant positive correlation between low network smoothness in class and distress among students (β = 0.767; *p* < 0.05). The worse the network smoothness, the more distressed they were, and vice versa. Students’ ability to adapt to the online teaching mode also significantly affected their distress. The greater their ability to adapt, the less likely they were to be distressed (β = −1.657; *p* < 0.001). In addition, there was a significant positive correlation between the economic pressure of online learning and students’ distress (β = 1.774; *p* < 0.05). If participating in online learning increased the pressure on their families, their distress also increased accordingly. The lower the burden, the lower the corresponding distress. This shows that Hypothesis 3 is confirmed.

Finally, the results of Models 1–4 show that the length of time was not associated with international students’ psychological distress. Moreover, there was no effect of age or gender on the psychological distress experienced by international students.

### 3.3. Robustness Analysis

**Figure 2:**
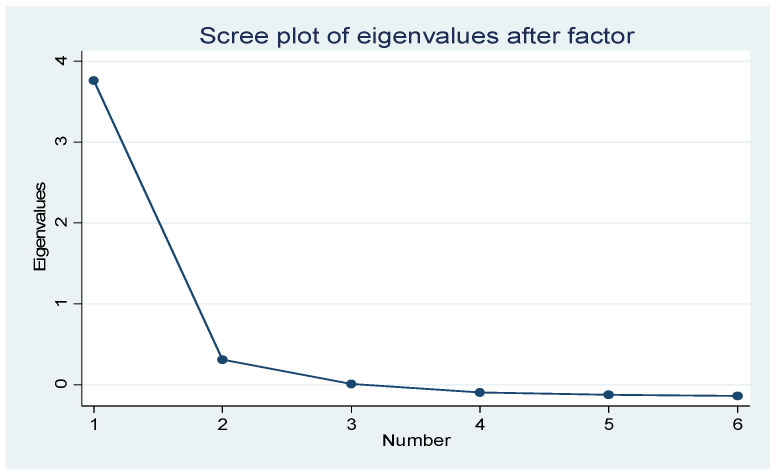
Scree plot of eigenvalues after factor.

In order to test the robustness of the above analytical results, we used another method to construct the dependent variable, psychological distress, and then rebuilt the models again. We sought to extract the factors associated with the six items of the scale by using exploratory factor analysis. The previous analysis showed that the reliability was good and the internal consistency was also high (Cronbach’s alpha = 0.9089), and the Kaiser–Meyer–Olkin value was 0.8744 (*p* < 0.000), which indicated that these items were suitable for exploratory factor analysis. Therefore, we conducted exploratory factor analysis by principal component and varimax rotation. From the results of Table 4 and Figure 2, we can see that only the eigenvalue of factor 1 was larger than 1, which shows that only one factor could be extracted. Thus, we extracted this factor and referred to it as psychological distress according to the correlation of the indicators and their meanings. The factor score was saved as the dependent variable.

With the same purpose as the previous four models, to detect the impacts of the environment, social support, values, and individual learning behaviors on psychological distress, as well as to confirm the robustness of the previous results, we established another four models, Models 5–8.

We rebuilt all the models (5–8) as in the previous analysis (Table 5), and all the results were consistent with those of the former models. This confirmed again that as long as there was sufficient formal social support, implementing social distancing early or later was not necessarily directly related to the psychological distress experienced by students. In addition, informal social support differed from formal social support during the COVID-19 epidemic, and it could significantly reduce international students’ psychological distress. Moreover, individualist values had a positive relationship with psychological distress, while collectivist values had the opposite relationship. Furthermore, new learning models and behaviors significantly correlated with psychological distress; specifically, low network smoothness and higher economic pressure all increased international students’ psychological distress. A better ability to adapt to the new online classes led to less psychological distress. These results confirmed the former analysis and showed that the results were robust.

## 4. Discussion

All the results showed that sex and age did not influence international students’ psychological distress, which is inconsistent with previous research [35,36,37]. The reason that there was no difference with regard to age and sex was probably that, in the face of a sudden epidemic, such as COVID-19, everyone is equally affected, regardless of their age or gender.

The above result shows that as long as there was sufficient social support, starting social distancing early or late was not necessarily directly related to the psychological distress experienced by students. There appears to be a significant gap between this result and general expectations. Typically, it is expected that community closures and social distancing will have a certain negative impact on individuals’ mental health status [57,58], because the closure will reduce the frequency of their face-to-face social interactions, and maintaining social distancing may reduce the quality of social interactions, which in turn can decrease or reduce the social support that individuals receive. However, the survey data show that the frequency and quality of international students’ social interactions in China were reduced due to the impact of the epidemic, while their mental health status was not significantly affected, which implies that they used new means to construct or maintain their social support.

The social support received by international students in China includes not only informal support from relatives and friends from home, other countries, and China, but also support from formal organizations such as Chinese universities. We can see that formal support can significantly reduce the psychological distress experienced by international students, but informal support does not show such an effect.

This conclusion may contradict previous conclusions about the influence of social support on mental health. Many previous studies have agreed that social support, especially informal social support, such as material or emotional support, has a positive effect on people’s mental health [21,22,59]. However, these studies were carried out under normal circumstances and could not take into account unusual circumstances, such as the emergence of new infectious diseases such as COVID-19. This study compensates for this gap in the existing research and examines the relationship between social support and psychological distress during the COVID-19 pandemic. This research reveals that, under unusual circumstances, the positive impact of informal social support on mental health may disappear, while formal social support still has a positive effect. Formal social support from the organizational level and the government could effectively alleviate the psychological distress caused by the COVID-19 epidemic. This conclusion will contribute to understanding the mechanism behind social support more deeply.

Moreover, different values significantly affected the psychological distress experienced by international students, which is partially consistent with previous research conclusions [27,28,60,61]. Although, in the initial stage, a lack of experience in responding to situations such as the COVID-19 pandemic caused certain problems in China, such as rapid spread, insufficient medical resources, and delayed treatment, it is undeniable that the epidemic in China has been effectively controlled in a relatively short period. Of course, this is closely related to China’s highly regulated, nationwide system, but the importance of the government’s control measures being implemented effectively and thoroughly should also be noted. More important is that most people respect the value of collectivism; upholding this value enables them to give way to the needs of the collective in the event of a major emergency, and to prioritize the interests of the collective over the individual so that the government’s decision-making can be implemented effectively in a short time, thus laying the foundation for overcoming the COVID-19 epidemic.

On the contrary, in western, developed countries, where liberal thought prevails, one of the important reasons that it is difficult to achieve similar results in a short period is probably that, under the guidance of free thought, individualism is valued above all, and citizens do not easily disregard their personal interests for collective interests. In the face of the COVID-19 epidemic, a major, rapidly emerging disease, it was necessary for governments to allocate resources in many cases, and individualism may lead to a resistance to unified deployment and command because this may well violate individual interests, thereby slowing down and reducing the efficiency of the governmental response to the epidemic; thus, the effectiveness of the response is greatly reduced.

In this sense, those who uphold the values of collectivism are likely to obey the government’s arrangements, adopt a positive attitude towards the government’s response to the epidemic, and believe that the government can utilize collective resources in order to control the epidemic; thus, such people will not experience significant anxiety due to the spread of the epidemic. On the contrary, those who uphold individualistic values are likely to resist the government’s allocation of resources and not obey the government’s arrangements, which will lead to the government’s inability to effectively use collective resources and extend the time needed for the government to control the epidemic. Furthermore, people who uphold individualistic values may be pessimistic about the process of the epidemic and the future of the epidemic, leading to a higher likelihood of experiencing anxiety and distress.

One of the main tasks of international students is to learn. However, due to the impact of the COVID-19 epidemic, almost all universities in China have been unable to carry out normal teaching activities by the conventional model, instead adopting an online teaching model. This new teaching model has been a major challenge for both the teachers and the students attending the class. Teachers have had to learn how to teach online, while students needed to adapt to the new model of learning alone, facing a computer screen. This brand-new model of teaching and learning means that a great deal of time is needed among teachers and students to learn run-in and debugging, so it is understandable that international students experienced difficulties adapting to this new mode, resulting in psychological distress. The main reason for such difficulties was poor adaptation to the new learning mode [24,62], and instead over-indulging on the internet [25], which can lead to offline communication obstacles.

Whether teachers and students can adapt to this model in a short time can determine the effect of teaching and learning. Moreover, the transition from traditional offline teaching to online teaching requires the purchase of certain equipment, such as computers, tablets, and mobile phones, for class, which may place additional financial pressure on students and their families, leading to psychological distress and even anxiety. At the same time, even if they possess such equipment, they had to ensure that their network was relatively smooth in order to achieve better learning results. If students experienced problems with their equipment or the class network, this greatly affected the normal class order and, ultimately, the learning effect, leading to their inability to complete the learning task satisfactorily and causing mental tension or anxiety.

The psychological distress caused by COVID-19 makes people feel restless, nervous, hopeless, and sad, which will probably induce anxiety. Although there is a significant difference between occasional and pathological anxiety, COVID-19 is not going to disappear suddenly for several years, in which there will still be the danger of infection, and this is when pathological COVID-19 anxiety will occur. Just as Tyrer comments that the vicious influence of the Internet and social media in promoting fear of disease may be the reason for the rise in pathological health anxiety in recent years, therefore, he suggests that people should follow his advice that limits unnecessary contact with health professionals of all types, and only listen to the news for a short time each day before the arrival of a vaccine [63]. At present, various vaccines have been developed, but the protective effect of vaccines is still controversial, and people’s worries still exist. Perhaps people need to adjust their mentality and correctly understand the controllable negative response of COVID-19 and its long-term coexistence with humans to reduce anxiety.

## 5. Conclusions

The psychological distress experienced by international students is the result of a combination of the external environment (including lockdown, social distancing, and social support) and internal factors such as values and behavior. The new teaching mode and the corresponding changes in learning behavior have been significantly associated with psychological distress brought about by the COVID-19 epidemic. Moreover, the influence of international students’ values also plays a significant role in their psychological distress. At the same time, this study also reveals that, if there is sufficient formal social support, quarantine or social distancing measures, implemented early or later, are not necessarily directly related to their psychological distress. The main contribution of this research is that, first of all, we overcame the single paradigm of psychological analysis, instead analyzing it from a multi-dimensional perspective. It is found that the psychological distress experienced by international students during the COVID-19 epidemic was not only affected by individual learning and other conditions, but also deeply affected by their values. Further analysis found that the values of collectivism helped the government to take emergency measures to effectively respond to the epidemic, thereby alleviating the psychological distress experienced by international students to a certain extent, while the values of individualism had no similar effect. Second, the research also finds that maintaining social distancing early or later was not necessarily closely related to psychological distress. The formal social support received by the individual played an important role and thereby alleviated their psychological distress, while informal support plays a small role.

## 6. Limitations

Moreover, it is important to recognize that the survey data sample of this study was derived only from an eastern university and may not be universal to a certain extent due to its size. Simultaneously, in order to collect data quickly, this study adopted an online questionnaire survey, which has its own shortcomings, such as sample selection bias and response rate problems. If future research can be sampled in a larger range and include more universities with strict sampling, this will not only be effective to improve the representativeness of the data but also make it possible to perform comparisons between international students from different countries, to help us to understand the general and specific causes of psychological distress; furthermore, this would also help us to further understand the formation mechanisms behind international students’ psychological distress.

## 7. Policy Implications

Closure and maintaining social distancing are effective ways to stop the spread of a virus. China has achieved good results throughout society (including universities) through these two methods. Although Chinese international students have experienced lockdowns and social distancing, their psychological distress is not severe. An important reason for this is that Chinese universities have provided sufficient formal social support to international students and received full cooperation from international students. This highlights that if other countries can provide sufficient formal social support, similar to China, then measures such as closing the community and maintaining social distancing can be adopted on a large scale. This will not necessarily cause psychological distress among citizens but could effectively mitigate the spread of the virus.

People who uphold individualistic values and collectivist values have not only experienced very different mental states during the COVID-19 epidemic, but they have also shown significant differences in cooperating with the government’s anti-epidemic actions. Research from China shows that, under such unique circumstances, individuals temporarily abandon their interests and obey the government’s arrangements to protect the interests of everyone to the greatest extent. On the contrary, if they uphold their individual interests, they may cause damage to the collective interests. This means that individual interests cannot be excessively advocated in the daily life process, and it is still necessary to properly cultivate the collective consciousness of citizens.

Research also shows that, during the epidemic, almost all colleges and universities have switched to new online teaching models, and students have had to buy new equipment and acquire a high-speed internet service. This has not only increased the financial pressure on international students but may also have prevented them from adapting to the new online teaching model quickly and effectively in a short period. This has greatly affected their psychological distress to a certain extent. Therefore, each university should take into account the difficulties that the new teaching model will bring to students, and try their best to provide financial and learning assistance to students to a certain extent. It should also be noted that the change in teaching mode during the COVID-19 epidemic may herald a major change in teaching modes in the future.

Given that psychological distress may be transformed into anxiety, psychiatrists and researchers have proposed clinical coping strategies [11,64,65,66]. These strategies are as follows: Firstly, to break isolation and attain social support—not only to increase communication with family members and friends, but also to strengthen contact and communication with formal organizations. Secondly, to rely on a limited amount of official information sources only and exclude unofficial channels and uncontrolled sources. Thirdly, to ask for professional help if the effects of stress are becoming too invasive. Finally, since COVID-19 is likely to coexist with humans for a long time, it is also necessary to maintain a good mentality while doing a good job of protection.

## Figures and Tables

**Table 1:** Description of the data.

	Frequency	Percentages	Mean Distress	
Sex				*p* > 0.05
male	177	61.25	13.70	
female	112	38.75	14.41	
Age group				*p* > 0.05
less than 23 years old	105	36.33	14.52	
23–30 years old	160	55.36	13.81	
31–36 years old	21	7.27	12.90	
37 years old and above	3	1.04	11.33	
Lockdown				*p* > 0.05
yes	274	94.81	13.91	
no	15	5.19	15.13	
Network smoothness				*p* < 0.05
very smooth	22	7.61	12.23	
smooth	76	26.30	12.01	
general	83	28.72	14.12	
occasionally stuck	48	16.61	16.71	
always stuck	15	5.19	17.00	
Adaptation to online class				*p* < 0.05
fully adapted	34	11.76	10.71	
more adapted	71	24.57	12.49	
about average	88	30.45	14.41	
not quite adapted	46	15.92	17.22	
not adapted	5	1.73	20	
Economic pressure				*p* < 0.05
no, opposite	6	2.46	12.83	
not at all	139	56.97	12.58	
yes, a little bit	99	40.57	16.01	
	**Mean**	**Std Dev**		
Length	27.7	30.90		
Distress score (sum)	13.97	6.38		
Support from their own country	1.36	0.54		
Support from other countries	1.19	0.60		
Support from Chinese classmates and friends	1.00	0.70		
Support from the university and faculties	1.31	0.58		
Time to start social distancing	1.84	1.02		
Values	4.70	1.42		

**Table 2:** Correlation matrix.

	Distress	Length	Support from Their Own	Support from Other Countries	Support from Chinese Classmates and Friends	Support from the University and Faculties	Network Smoothness	Adaptation to Online Classes	Economic Pressure	Values	Time to Start Social Distancing
Distress	1										
Length	0.0307	1									
Support from their own country	−0.0980	0.1192	1								
Support from other countries	−0.0781	−0.0150	0.3485 ***	1							
Support from Chinese classmates and friends	−0.0328	0.0789	0.2025 ***	0.4758 ***	1						
Support from the university and faculties	−0.1615 *	0.1039	0.3339 ***	0.4405 ***	0.4021 ***	1					
Network smoothness	0.1061 *	−0.088	−0.0851	−0.0541	−0.0407	0.0284	1				
Adaptation to online classes	0.1309 **	−0.0924	−0.0946	−0.046	−0.0641	0.0284	0.8907 **	1			
Economic pressure	0.2461 **	−0.0948	−0.0751	0.003	−0.0370	−0.0995	0.1843 **	0.1428 **	1		
Values	0.2657 **	0.1191	0.0164	0.0097	0.0559	−0.0901	−0.1152	−0.1256	0.1533 **	1	
Time to start social distancing	0.0829	0.0882	−0.0329	−0.0401	0.0682	0.0261	0.0384	0.0597	0.0595	0.1502 **	1

* *p* < 0.05, ** *p* < 0.01, *** *p* < 0.001.

**Table 3:** Regression model of lockdown, social support, and learning behavior (distress).

	Model 1	Model 2	Model 3	Model 4
Sex (Male = 0)	0.610	0.730	0.509	1.198
	(0.784)	(0.781)	(0.762)	(0.786)
Age group (less than 23 = 0)				
23–30 years old	−0.660	−0.844	−0.694	0.268
	(0.811)	(0.807)	(0.787)	(0.804)
31–36 years old	−1.406	−1.089	−0.135	0.437
	(1.556)	(1.548)	(1.526)	(1.549)
Above 37 years old	−3.115	−2.276	−1.855	3.708
	(3.756)	(3.732)	(3.636)	(5.923)
Length of stay in China	0.005	0.009	0.003	0.005
	(0.012)	(0.012)	(0.012)	(0.013)
Lockdown (No = 0)	0.833	0.976	0.762	−0.900
	(1.727)	(1.714)	(1.670)	(1.773)
Time to start social distancing	0.476	0.469	0.278	0.407
	(0.378)	(0.376)	(0.370)	(0.383)
Support from their own		−0.661	−0.761	−0.497
		(0.764)	(0.744)	(0.769)
Support from other countries		−0.037	−0.156	−0.284
		(0.780)	(0.760)	(0.752)
Support from Chinese classmates and friends		0.391	0.214	0.245
		(0.637)	(0.621)	(0.655)
Support from the university and faculties		−1.841 *	−1.410	−2.001 *
		(0.761)	(0.748)	(0.795)
Values			1.076 ***	0.826 **
			(0.268)	(0.281)
Poor smoothness of internet				0.767 *
				(0.389)
Adaptation to online classes				−1.657 ***
				(0.405)
Economic pressure				1.774 *
				(0.729)
	13.18 ***	16.05 ***	11.34 ***	1.251
Constant	(1.036)	(1.523)	(1.889)	(2.729)
N	289	289	289	244
R^2^	0.011	0.046	0.082	0.259

* *p* < 0.05, ** *p* < 0.01, *** *p* < 0.001.

**Table 4:** Exploratory factor analysis of distress.

Variable	Factor 1	Factor 2	Factor 3	Uniqueness
feel so sad that nothing could cheer you up	0.8148	−0.1920	−0.0525	0.2965
feel nervous	0.7835	−0.3185	0.0145	0.2845
feel so restless that you could not sit still	0.8282	−0.1188	0.0628	0.2961
feel hopeless	0.7173	0.2746	0.0359	0.4088
feel that everything was an effort	0.809	0.1249	−0.0523	0.3273
feel worthless	0.7946	0.2597	−0.0051	0.3012

**Table 5:** Regression model of lockdown, social support, and learning behavior (factor).

	Model 5	Model 6	Model 7	Model 8
Sex (Male = 0)	0.078	0.096	0.063	0.165
	(0.117)	(0.117)	(0.114)	(0.117)
Age group (less than 23 = 0)				
23–30 years old	−0.092	−0.119	−0.097	0.044
	(0.121)	(0.121)	(0.118)	(0.120)
31–36 years old	−0.221	−0.173	−0.032	0.054
	(0.233)	(0.231)	(0.228)	(0.231)
Above 37 years old	−0.460	−0.333	−0.271	0.545
	(0.562)	(0.558)	(0.544)	(0.884)
Length of stay in China	0.001	0.001	0.001	0.001
	(0.002)	(0.002)	(0.002)	(0.002)
Lockdown (No = 0)	0.125	0.147	0.116	−0.135
	(0.258)	(0.256)	(0.250)	(0.265)
Time to start social distancing	0.069	0.069	0.040	0.061
	(0.057)	(0.056)	(0.055)	(0.057)
Support from their own		−0.106	−0.121	−0.0797
		(0.114)	(0.111)	(0.115)
Support from other countries		−0.007	−0.024	−0.043
		(0.117)	(0.114)	(0.112)
Support from Chinese classmates and friends		0.056	0.029	0.032
		(0.0951)	(0.093)	(0.098)
Support from the university and faculties		−0.275 *	−0.211	−0.300 *
		(0.114)	(0.112)	(0.119)
Values			0.158 ***	0.121 **
			(0.040)	(0.042)
Poor smoothness of internet				0.116 *
				(0.058)
Adaptation to online classes				−0.249 ***
				(0.060)
Economic pressure				0.279 *
				(0.109)
Constant	−0.115	0.327	−0.366	−1.918 ***
	(0.155)	(0.228)	(0.283)	(0.407)
N	289	289	289	244
R^2^	0.017	0.049	0.104	0.268

* *p* < 0.05, ** *p* < 0.01, *** *p* < 0.001.

## Data Availability

The data presented in this study are available on request from the author.
